# Supplementary material for: Sorting of a multi-subunit ubiquitin ligase complex in the endolysosome system
Source: eLife. 2018 Jan 22;7:e33116. doi: 10.7554/eLife.33116 (PMC5811209; doi:10.7554/eLife.33116)
Supplement: Supplementary file 1. [file elife-33116-supp1.docx]

**Supplemental Data**

Sorting of a Multi-subunit Ubiquitin Ligase Complex in the Endolysosome System

Xi Yang, Felichi Mae Arines, Weichao Zhang, and Ming Li

**Supplemental File 1:**

| **Supplemental Table 1: Yeast strains and Plasmids used in this study** | | | | |
| --- | --- | --- | --- | --- |
| *S. cerevisiae strains* | | | | |
| ***strain*** | ***name*** | ***genotype*** | ***reference/source*** | |
| SEY6210 | wild type | Matα*, leu1-3, 112 ura3-52 his3-200, trp1-901 lys2-801 suc2-D9* | (Robinson et al., 1988) | |
| SEY6210.1 | wild type | Mata*, leu1-3, 112 ura3-52 his3-200, trp1-901 lys2-801 suc2-D9* | (Robinson et al., 1988) | |
| BY4742 | wild type | Matα,*his3*Δ1,*leu2Δ0,lys2Δ0,ura3Δ0* | Invitrogen | |
| YXY189 | Ubx3-nG,Vph1-mCh | 6210.1, UBX3-neonGreen-3HA::HIS3, VPH1-mCherry::TRP1 | This study | |
| YXY216 | Ubx3-nG,Vph1-mCh,*vps27*Δ | 6210.1, VPH1-mCherry::KAN,  *vps27*Δ::HIS3,  UBX3-neonGreen::TRP1 | This study | |
| YXY221 | Ubx3-nG,Vph1-mCh,*pep12*Δ | 6210.1, VPH1-mCherry::KAN,  UBX3-neonGreen-3HA::HIS3,*pep12*Δ::TRP1 | This study | |
| YXY219 | Ubx3-nG,Vph1-mCh,*apl6*Δ | 6210.1, VPH1-mCherry::HIS3,*apl6*Δ::KAN,UBX3-neonGreen::TRP1 | This study | |
| YML100 | *pep4*Δ | 6210, *pep4*Δ::LEU2, | (Li et al., 2015) | |
| YML499 | Ubx3-Flag | 6210, *pep4*Δ::LEU2, UBX3-HTF::HIS3 | (Li et al., 2015) | |
| YXY032 | 014w-Flag | 6210, YIR014W-HTF::HIS3, *pep4*Δ::LEU2 | This study | |
| YXY092 | 109w-Flag | 6210, YPR109W-HTF::HIS3, *pep4*Δ::LEU2 | This study | |
| YXY088 | 014w-HA,109w-Flag | 6210, YIR014W-3HA::TRP1, YPR109W-HTF::HIS3, *pep4*Δ::LEU2 | This study | |
| YXY025 | Vld1-nG | 6210.1, VLD1-neonGreen-3HA::TRP1 | This study | |
| YXY098 | Gld1-nG | 6210.1, GLD1-neonGreen-3HA::TRP1 | This study | |
| FAY003 | Mars-Sec7,Gld1-nG | 6210.1, GLD1-neonGreen-3HA::HIS3, MARS-SEC7::TRP1 | This study | |
| YML1133 | FRB-mCh,  Ubx3-nG,  Gld1-FKBP | 6210.1, *tor1-1*, *frb1*Δ::NAT, UBX3-neonGreen::KAN, GLD1-2xFKBP::TRP1, PRS306-Pssh4-Frb-mCherry-NLS-T_ADH1_ | This study | |
| YML553 | Ubx3-nG | 6210.1, UBX3-neonGreen::TRP1 | This study | |
| YXY042 | Ubx3-nG, *vld1*Δ | 6210.1, UBX3-neonGreen-3HA::HIS3,*vld1*Δ::TRP1 | This study | |
| YXY055 | Ubx3-nG, *gld1*Δ | 6210.1, UBX3-neonGreen-3HA::HIS3,*gld1*Δ::TRP1 | This study | |
| YXY022 | yir014wΔ | 4742, *yir014wΔ*::KAN | Invitrogen Mat-Alpha DEL Complete Set (Cat. no. 95401.H3) | |
| YXY143 | ypr109wΔ | 4742, *ypr109w*Δ::KAN | Invitrogen Mat-Alpha DEL Complete Set (Cat. no. 95401.H3) | |
| YXY010 | *tul1*Δ | 4742, *tul1*Δ::KAN | Invitrogen Mat-Alpha DEL Complete Set (Cat. no. 95401.H3) | |
| YXY047 | Vld1-nG,Vph1-mCh | 6210.1, VPH1-mCherry::HIS3,Vld1-neonGreen-3HA::TRP1 | This study | |
| YXY215 | Vld1-nG,Vph1-mCh,*pep12*Δ | 6210.1, VPH1-mCherry::KAN,Vld1-neonGreen-3HA::HIS3,*pep12*Δ::TRP1 | This study | |
| YXY218 | Vld1-nG,Vph1-mCh,*vps27*Δ | 6210.1, VPH1-mCherry::KAN,  *vps27*Δ::HIS3,VLD1-neonGreen-3HA::TRP1 | This study | |
| YXY220 | Vld1-nG,Vph1-mCh,*apl6*Δ | 6210.1, VPH1-mcherry::HIS3*,apl6Δ*::KAN,Vld1-neonGreen-3HA::TRP1 | This study | |
| YXY058 | Vld1^Δ6AA^-nG | 6210.1, VLD1^(Δ6AA)-^neonGreen-3HA::HIS3 | This study | |
| YXY059 | Vld1^Δ6AA^-nG,*pep12*Δ | 6210.1, VLD1^(Δ6AA)-^neonGreen-3HA::HIS3,*pep12*Δ::TRP1 | This study | |
| YXY194 | Gld1-nG,Vph1-mCh | 6210.1, VPH1-mCherry::KAN, GLD1-neonGreen-3HA::TRP1 | This study | |
| YXY193 | Gld1-nG,Vph1-mCh,*vps27*Δ | 6210.1, VPH1-mCherry::KAN,*vps27*Δ::HIS3, GLD1-neonGreen-3HA::TRP1 | This study | |
| YXY192 | Gld1-nG,Vph1-mCh,*pep12*Δ | 6210.1, VPH1-mCherry::KAN, GLD1-neonGreen-3HA::HIS3, *pep12*Δ::TRP1 | This study | |
| YXY090 | Gld1-nG,*vps35*Δ | 6210.1, *vps35*Δ::TRP, GLD1-neonGreen::HIS3 | This study | |
| YXY033 | Vld1-nG,*tul1*Δ | 6210.1, VLD1-neonGreen-3HA::HIS3, *tul1*Δ::TRP1 | This study | |
| YXY034 | Vld1-nG,*dsc2*Δ | 6210.1, VLD1-neonGreen-3HA::HIS3, *dsc2*Δ::TRP1 | This study | |
| YXY035 | Vld1-nG,*dsc3*Δ | 6210.1, VLD1-neonGreen-3HA::HIS3, *dsc3*Δ::TRP1 | This study | |
| YXY037 | Vld1-nG,*ubx3*Δ | 6210.1, VLD1-neonGreen-3HA::TRP1, *ubx3*Δ::HIS3 | This study | |
| YXY096 | Gld1-nG,*tul1*Δ | 6210.1, GLD1-neonGreen-3HA::HIS3, *tul1*Δ::TRP1 | This study | |
| YXY094 | Gld1-nG,*dsc2*Δ | 6210.1,GLD1-neonGreen-3HA::HIS3, *dsc2*Δ::TRP1 | This study | |
| YXY093 | Gld1-nG,dsc3Δ | 6210.1, GLD1-neonGreen-3HA::HIS3, *dsc3*Δ::TRP1 | This study | |
| YXY097 | Gld1-nG,ubx3Δ | 6210.1, GLD1--neonGreen-3HA::TRP1, *ubx3*Δ::HIS3 | This study | |
| YXY038 | Ubx3-nG,*tul1*Δ | 6210.1, UBX3-neonGreen-3HA::HIS3,*tul1*Δ::TRP1 | This study | |
| YXY039 | Ubx3-nG,*dsc2*Δ | 6210.1, UBX3-neonGreen-3HA::HIS3, *dsc2*Δ::TRP1 | This study | |
| YXY040 | Ubx3-nG,*dsc3*Δ | 6210.1, UBX3-neonGreen-3HA::HIS3, *dsc3*Δ::TRP1 | This study | |
| FAY001 | Ubx3-nG, *vld1*Δ,*gld1*Δ | 6210.1, UBX3-neonGreen-3HA::HIS3, *vld1*Δ::TRP1, *gld1*Δ::KAN | This study | |
| YXY115 | Vld1-nG, *tul1*Δ,*dsc3*Δ | 6210.1, *tul1*Δ::KAN, VLD1-neonGreen-3HA::HIS3, *dsc3*Δ::TRP1 | This study | |
| YXY116 | Gld1-nG, *tul1*Δ,*dsc3*Δ | 6210.1, *tul1*Δ::KAN,GLD1-neonGreen-3HA::HIS3, *dsc3*Δ::TRP1 | This study | |
| YXY114 | Ubx3-nG, *tul1*Δ,*dsc3*Δ | 6210.1, *tul1*Δ::KAN, Ubx3-neonGreen::HIS3, *dsc3*Δ::TRP1 | This study | |
| YXY167 | Ubx3-Flag, Vld1-HA, Gld1-GFP | 6210,  VLD1-3HA::KAN,  GLD1-GFP::TRP1,UBX3-HTF::HIS3, *pep4*Δ::LEU2 | This study | |
| YXY184 | Ubx3-Flag, Vld1-HA, Gld1-GFP, *tul1*Δ, *dsc3*Δ | 6210, *dsc3*Δ::NAT,*tul1*Δ::HYG,VLD1-3HA::KAN,GLD1-GFP::TRP1, UBX3-HTF::HIS3, *pep4*Δ::LEU2 | This study | |
| YXY183 | Ubx3-Flag, Vld1-HA, Gld1-GFP, *dsc2*Δ | 6210, *dsc2*Δ::HYG, VLD1-3HA::KAN, GLD1-GFP::TRP1, UBX3-HTF::HIS3, *pep4*Δ::LEU2 | This study | |
| YXY141 | Ubx3-Flag, *vld1*Δ, Gld1-GFP | 6210, *vld1*Δ::KAN, GLD1-GFP::TRP1, UBX3-HTF::HIS3, *pep4*Δ::LEU2 | This study | |
| YXY185 | Ubx3-Flag, Vld1-HA, *gld1*Δ | 6210, *gld1*Δ::TRP1, VLD1-3HA::KAN, UBX3-HTF::HIS3, *pep4*Δ::LEU2 | This study | |
| YXY181 | Ubx3-Flag, *vld1*Δ, *gld1*Δ | 6210,*gld1*Δ::TRP1, *vld1*Δ::KAN,UBX3-HTF::HIS3, *pep4*Δ::LEU2 | This study | |
| *S. cerevisiae expression plasmids* | | | | |
| ***vector*** | ***name*** | ***description*** | | ***reference/source*** |
| pRS416 | DsRed-HDEL | GPD promoter N-terminal DsRed | | This study |
| pRS415 | pVLD1-VLD1 | endogenous promoter | | This study |
| pRS415 | pGPD-VLD1 | GPD promoter | | This study |
| pRS415 | pGLD1-GLD1 | endogenous promoter | | This study |
| pRS415 | pGPD-GLD1 | GPD promoter | | This study |
| pRS416 | GFP-YIF1 | ADH1 promoter, N-terminal GFP | | This study |
| pCM189 | COT1-GFP | Tet-off vector, tet-O7 promoter C-terminal GFP | | Li et al., 2015 |
| pRS416 | pGPD-VLD1-HA | GPD promoter | | This study |
| pRS416 | pGPD-GLD1-GFP | GPD promoter | | This study |
| pCM189 | pCM189-pYPQ1- GFP-YIF1 | Tet-off vector, tet-O7+ YPQ1 promoter N-terminal GFP | | This study |
| pRS415 | pADH1-*sp*DSC4 | ADH1 promoter | | This study |
